# Biased gene expression reveals the contribution of subgenome to altitude adaptation in allopolyploid *Isoetes sinensis*

**DOI:** 10.1002/ece3.9677

**Published:** 2022-12-28

**Authors:** Pei Wei, Xiao‐lei Yu, Yu‐jiao Yang, Zhu‐yifu Chen, Shu‐qi Zhao, Xin‐zhong Li, Wen‐cai Zhang, Chen‐lai Liu, Xiao‐yan Li, Xing Liu

**Affiliations:** ^1^ State Key Laboratory of Hybrid Rice, Laboratory of Plant Systematics and Evolutionary Biology, College of Life Sciences Wuhan University Wuhan China; ^2^ Laboratory of Extreme Environmental Biological Resources and Adaptive Evolution, Research Center for Ecology, School of Sciences Tibet University Lhasa China; ^3^ Biology Experimental Teaching Center, School of Life Science Wuhan University Wuhan China

**Keywords:** adaptation, allopolyploid, expression pattern, *Isoetes sinensis*, transplant

## Abstract

Allopolyploids are believed to inherit the genetic characteristics of its progenitors and exhibit stronger adaptability and vigor. The allotetraploid *Isoetes sinensis* was formed by the natural hybridization and polyploidization of two diploid progenitors, *Isoetes taiwanensis* and *Isoetes yunguiensis*, and was believed to have the potential to adapt to plateau environments. To explore the expression pattern of homoeologous genes and their contributions to altitude adaptation, we transplanted natural allotetraploid *I. sinensis* (TnTnYnYn) along the altitude gradient for a long‐term, and harvested them in summer and winter, respectively. One year after transplanting, it still lived well, even in the extreme environment of the Qinghai‐Tibet Plateau. Then, we performed high‐throughput RNA sequencing to measure their gene expression level. A total of 7801 homoeologous genes were expressed, among which 5786 were identified as shared expression in different altitudes and seasons. We further found that altitude variations could change the subgenome bias trend of *I. sinensis*, but season could not. Moreover, the functions of uniquely expressed genes indicated that temperature might be an important restrictive factor during the adaptation process. Through the analysis of DEGs and uniquely expressed genes, we found that Y subgenome provided more contributions to high altitude adaptation than T subgenome. These adaptive traits to high altitude may be inherited from its plateau progenitor *I. yunguiensis*. Through weighted gene co‐expression network analysis, pentatricopeptide repeats gene family and glycerophospholipid metabolism pathway were considered to play important roles in high‐altitude adaptation. Totally, this study will enrich our understanding of allopolyploid in environmental adaptation.

## Graphical abstract

Long‐term transplanting experiment was performed to simulate real local habitats. The genomes of *Isoetes taiwanensis* and *Isoetes yunguiensis* (diploid progenitors) were used as references to separately obtain the two subgenomes transcriptome expression data of *Isoetes sinensis* (subgenome T and Y).Through transcriptomic analysis, we demonstrated that the potential adaptation of *I. sinensis* to high altitude inherited from its plateau progenitor, *I. yunguiensis*.

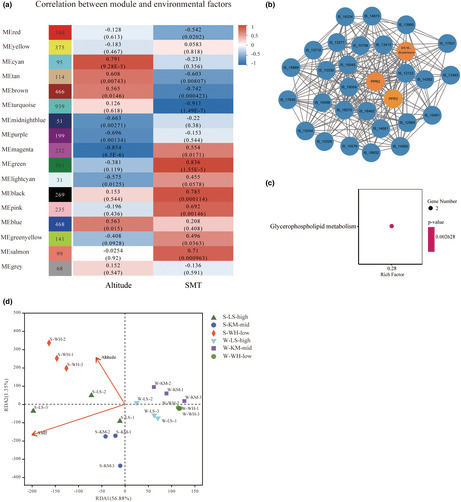

## 1 INTRODUCTION

Widespread in plants, polyploidization is an important force in plant evolution (Soltis et al., 2015). Polyploidy is mainly divided into two types: allopolyploidy and autopolyploidy. Allopolyploids originate from intergeneric or interspecific hybridization which consist of two or more divergent homoeologous genomes, whereas autopolyploids originate from intraspecific genome duplication which consist of multiple sets of similar or identical genomes (Doyle et al., 2008). Compared with autopolyploid, allopolyploid has greater vigor and adaptability and is considered to play a significant role in the diversification and speciation of plants (Abbott et al., 2013; Chen, 2007; Li et al., 2014). Previous studies focused on some important crops, such as rapeseed (*Brassica napus*), tobacco (*Nicotiana tabacum*), wheat (*Triticum aestivum*), and cotton (*Gossypium hirsutum*), in which homoeolog expression bias and expression level dominance have been well studied. However, few studies have focused on the effects of environmental factors on the expression of homoeologous genes. Even though there are some controlled experiments involving the response of homoeologous gene expression to environmental temperature (Combes et al., 2012, 2013), the expression pattern of homoeologous gene to the natural environment is still unknown.

**FIGURE 1:**
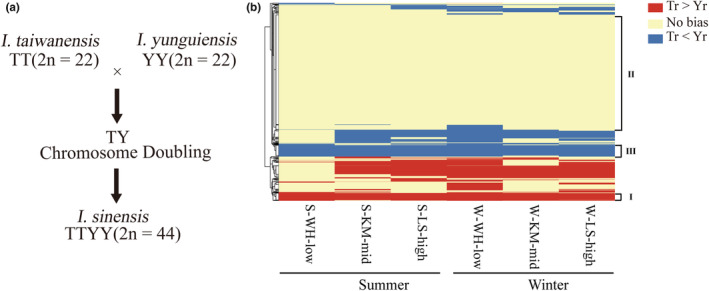
(a) The origin of allotetraploid *Isoetes sinensis*. (b) Heatmap of shared homologous gene pairs expressed at three altitudes in two seasons. The red area indicates bias toward T subgenome, blue bias toward Y subgenome, and yellow no bias.

*Isoetes* L. is an ancient heterosporous lycopsid which can be traced back to the Devonian Period. There are 150–300 extant species of *Isoetes* L. which are widely distributed around the world (Pigg, 2001; Taylor & Hickey, 1992). In China, there are a total of 6 *Isoetes* species distributed at different altitudes. Diploids are mainly distributed in plateau areas except for *I. taiwanensis*. Among them, *I. shangrilaensis* and *I. hypsophila* occur at the Qinghai‐Tibet Plateau (QTP), while *I. yunguiensis* occurs at the Yunnan‐Guizhou Plateau (YGP) (Li et al., 2019; Liu et al., 2004). Moreover, two polyploid species, *I. sinensis* (4*n* = 44) and *I. orientalis* (6*n* = 66), occur at low‐altitude area on the Middle‐lower Yangtze Plain (MYP) (Hong & Taylor, 2005). The allotetraploid *I. sinensis* was formed by the natural hybridization and polyploidization of *I. taiwanensis* and *I. yunguiensis* (Figure 1a) (Taylor et al., 2004). A previous study proposed the hypothesis that *I. sinensis* had inherited some abilities from its plateau progenitor *I. yunguiensis* to adapt to the plateau, and it could thrive on plateaus (Dai et al., 2021). However, whether these abilities are really inherited from its plateau progenitor remains to be further verified.

Transplanting experiment is a direct and powerful way to study how plants will respond to environmental change (Nooten & Hughes, 2017). In general, transplanting will cause damage to the shoots or roots (Salam et al., 2001; Sasaki & Gotoh, 1999). Some previous studies have demonstrated that transcriptome shock occurred at the early stage of the transplanting experiment which could cause growth inhibition (Kotera et al., 2004; Torres et al., 1994). The effect caused by transplantation will decrease as time elapsed. In this study, we transplanted *I. sinensis* to three elevation gradients, a high‐altitude (QTP: Lhasa; 29°38′41″N, 91°10′43″E; alt. 3662 m), a mid‐altitude (YGP: Kunming; 25°03′26″N, 102°42′02″E; alt. 1905 m), and a low‐altitude (MYP: Wuhan; 30°32′17″N, 114°21′18″E; alt. 35 m). To avoid the interference caused by transplanting to our study, we choose to sample after 1 year. We sampled the fresh leaves during summer and winter. Then the RNA sequencing of all samples were performed. In short‐term adaptation, the regulation of gene expression can serve as a mechanistic link from genetic and cellular processes to the physiological responses that allow organisms to rapidly accommodate the changing environments (Rivera et al., 2021). In other words, the impact of environmental changes on the homoeologous expression will be revealed at transcriptome level. Here we performed comparative transcriptomic analysis to measure the contribution of T subgenome and Y subgenome to altitude adaptation. We also showed the influence of altitude and season on homoeologous expression patterns.

## 2 MATERIALS AND METHODS

### 2.1 Transplanting experiments

To explore the variations in the transcriptomic expression of allopolyploid *I. sinensis* among different altitudes and seasons, we collected natural individuals from Jiande city, Zhejiang province, and transplanted them along gradient altitudes: Wuhan, Kunming, and Lhasa. All individuals were planted in outdoor plots to simulate real local habitats. The environmental conditions of three transplant plots were consistent with the report of *Isoetes* species distributional pattern in China (Liu et al., 2004). To reduce the influence caused by transplanting, all samples were harvested from fresh leaves after at least 1 year. Long term locally planting could make transcriptome expression have a stable condition. And the sampling in winter and summer was on the same individual. After harvesting, the fresh leaves were dissected and frozen in liquid nitrogen, then stored at −80°C until being subjected to transcriptome sequencing. The Illumina HiSeq X Ten platform was used to generate 150‐bp paired‐end reads. Our study consisted of 6 groups: summer and winter of WH‐low, LS‐high and KM‐mid, respectively, each with three biological replicates. Summer sequencing data were obtained from previous study in our lab (Dai et al., 2021).

### 2.2 Mapping to reference genomes, normalization of transcriptome expression

To obtain clean reads, quality control and filtering of raw data were conducted by the software Fastp. *I. taiwanensis* genome (https://genomevolution.org/coge/GenomeInfo.pl?gid=61511) and *I. yunguiensis* (unpublished data) genome were integrated and used as reference genome in this study. The software HISAT2 was used to map the clean reads to reference genome (version 2.2.1) (Kim et al., 2015). Only the uniquely mapped reads were used for subsequent analysis. Then we used software featureCounts (Yang et al., 2014) to quantify the expression of genes. During this process, the GTF (gene transfer format) information of *I. yunguiensis* and *I. taiwanensis* was used in featureCounts, respectively; therefore, genes expression of T subgenome and Y subgenome were successfully distinguished. The method described above drew on previously published study of allotetraploid *Brassica napus* (Li et al., 2020). To reduce the transcriptional noise, we set a strict screening criterion. The gene expression level meeting at least two of the three biological replicates expressed more than 1 was used in our study. The expression level of genes was normalized by TMM (trimmed mean of *M*‐values). To further get the homoeologous gene expression, we used OrthoFinder (version 2.5.4) (Emms & Kelly, 2015) to identify 8739 homoeologous gene pairs across its two diploid progenitors, among which 7801 genes were expressed in different environmental conditions.

### 2.3 Identification of differentially expressed genes (DEGs) and gene annotation

DESeq2 method (version: 1.34.0) was used to identify DEGs between T and Y subgenome (Love et al., 2014). Genes meeting the criteria |log2foldchange| > 1 and adjusted *p* value (*p*adj) < .001 were identified as DEGs. All homoeologous genes were annotated by online website eggNOG‐mapper (version: 2.1.7; http://eggnog‐mapper.embl.de/) (Jaime, Kristoffer, et al., 2016) based on the eggNOG 4.5 orthology data (Jaime, Damian, et al., 2016). To study the contribution of different subgenomes to altitude adaptation, KEGG (Kyoto Encyclopedia of Genes and Genomes) enrichment analysis of the DEGs was performed on the OmicShare, a free online platform for data analysis (www.omicshare.com/tools).

### 2.4 Weighted gene co‐expression network analysis and selection of hub genes

To identify altitude‐related homoeologous genes, the *R* package weighted gene co‐expression network analysis (WGCNA) was used to construct homoeologous gene co‐expression networks for *I. sinensis*. Genes that clustered in modules were associated with altitude and seasonal mean temperature (SMT) (Langfelder & Horvath, 2008). The SMT was downloaded from ArcGIS database (Table S1). Hub genes were selected based on KME values, and visualized by the sofware Cytoscape (version 3.9.1) (Shannon et al., 2003).

## 3 RESULTS

### 3.1 Transcriptome sequencing and reads mapping

A total of 18 samples were used to construct libraries and conduct RNA sequencing. After filtering and quality control, a total of 824.69 million (M) clean reads were obtained from 834.87 million raw reads. All Q20 and Q30 percentages of 18 samples exceeded 96.79% and 91.80%, indicating that our sequencing data was of high quality for subsequent analysis. An average 63.46% of reads per sample were uniquely mapped to the integrated reference genome (Table S2). Most of the Pearson correlation coefficient (*R*) exceeded 0.90 indicating that the correlations of gene expression between three biological replicates of each group were high (Figure S1).

### 3.2 Overview of homoeologous gene expression patterns

We studied the shared and all homoeologous gene expression, respectively. Ultimately, a total of 5786 homoeologous gene pairs were identified as shared expression that expressed in all six different spatiotemporal conditions (Table 1). In summer, 749, 1528, and 1614 genes were identified as biased expression in WH‐low, KM‐mid, and LS‐high, while the corresponding numbers in winter were 2238, 1430, and 1729, respectively. The ratio of bias‐expressed gene ranged from 12.95% to 38.68%, and both the most and the fewest conditions were observed in WH‐low. Whatever it was summer or winter, *I. sinensis* transplanted in WH‐low and KM‐mid were always biased toward Y subgenome while biased toward T subgenome in LS‐high. However, statistical results indicated that the degree of all bias was very slight that most ratios of subgenome bias were in the range of 50% ± 2%.

**TABLE 1:** List of shared homoeologous genes

Season	Location	Shared homoeologous gene pairs	Total bias	Y‐biased	T‐biased	Bias/shared(%)	Significance^a^	Bias toward
Summer	S‐WH	5786	749	440	309	12.95	***	Y
	S‐KM	5786	1528	774	754	26.41	***	Y
	S‐LS	5786	1641	816	825	28.36	***	T
Winter	W‐WH	5786	2238	1122	1116	38.68	***	Y
	W‐KM	5786	1430	735	695	24.71	***	Y
	W‐LS	5786	1729	852	877	29.88	***	T

^a^ *p*‐adj value of DESeq2; *p‐adj < .05; **p‐adj < .01; ***p‐adj < .001.

Hierarchical clustering revealed different expression bias patterns, including genes having consistent bias, distinct bias to different environments, or no bias. For instance, areas I, III, and II showed consistent T‐biased, Y‐biased and no bias, respectively (Figure 1b). The heatmap analysis showed that a considerable number of homoeologous genes maintained their bias direction.

We further analyzed all homoeologous genes in six groups. In summer, 6445, 6295 and 6170 homoeologous genes were detected in WH‐low, KM‐mid, and LS‐high, while the corresponding counts in winter were 6434, 6568 and 6351, respectively. More detailed information was provided in Table 2. In summer, the number of biased genes seemed to increase with altitude (Table 2). However, this trend was not observed in winter, from which we found that WH‐low had more biased expression genes than KM‐mid and LS‐high in winter. The number of biased expression genes in WH‐low was significantly different in summer and winter (summer: 749; winter: 2238), possibly due to greater seasonal variation in WH‐low. Furthermore, the degree of all bias was also slight, which was consistent with the observation from the expression of shared homoeologous genes (Tables 1 and 2). Besides, we found that both shared and all homoeologous genes had a consistent tendency of subgenome bias (i.e., both shared and all homoeologous genes showed bias toward Y, Y, and T subgenome in summer and winter), which might be explained by a large proportion of shared homoeologous genes in all homoeologous genes.

**TABLE 2:** List of all homoeologous genes

Season	Location	All homoeologous gene pairs	Total bias	Y‐biased	T‐biased	Bias/all (%)	Significance^a^	Bias toward
Summer	S‐WH	6445	826	506	320	12.82%	***	Y
	S‐KM	6295	1573	795	778	24.99%	***	Y
	S‐LS	6170	1667	831	836	27.02%	***	T
Winter	W‐WH	6434	2412	1210	1202	37.49%	***	Y
	W‐KM	6568	1492	767	725	22.72%	***	Y
	W‐LS	6351	1776	880	896	27.96%	***	T

^a^ *p*‐adj value of DESeq2; *p‐adj < .05; **p‐adj < .01; ***p‐adj < .001.

### 3.3 Comparison of Subgenome‐Biased genes at different altitudes and seasons

**FIGURE 2:**
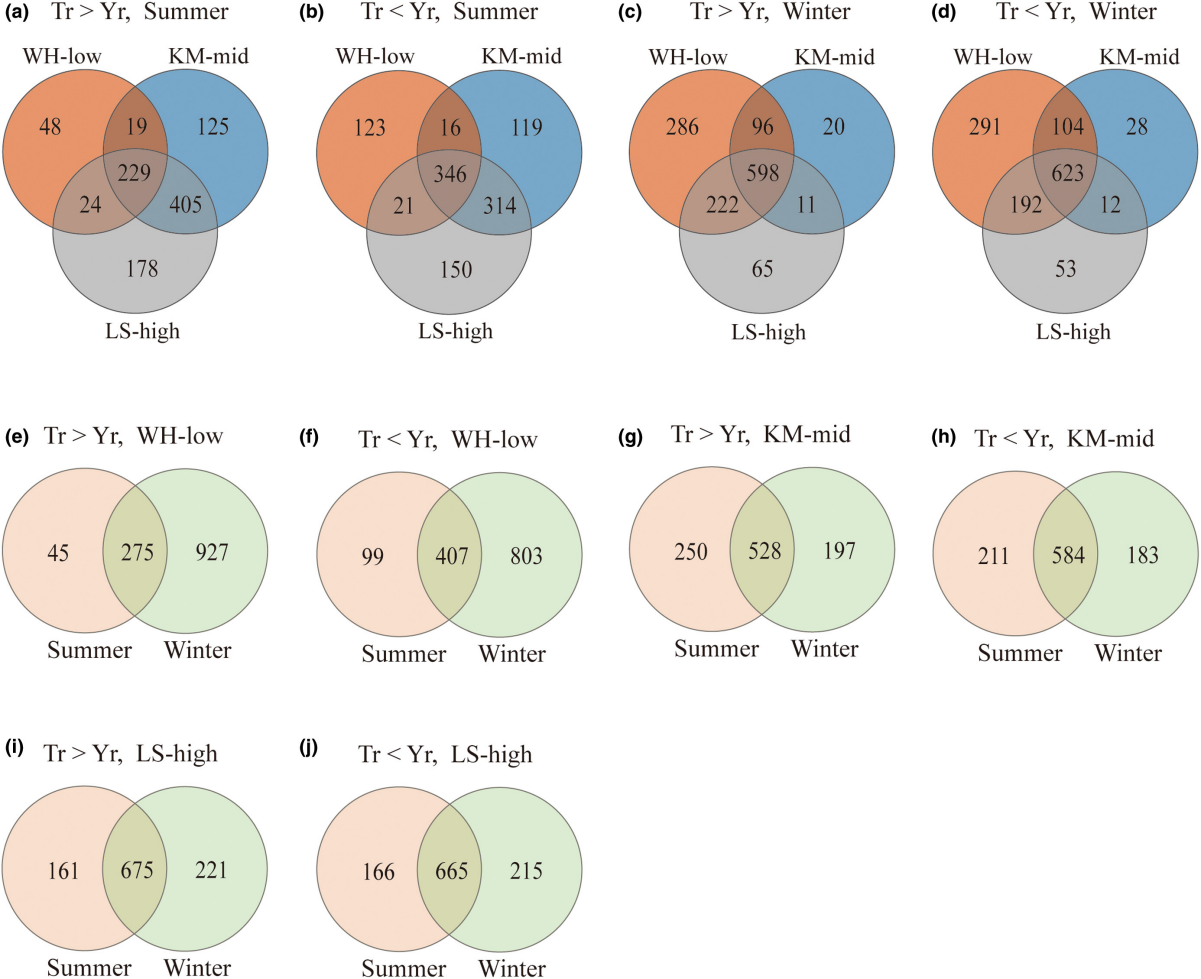
Comparison of subgenome‐biased genes. Each circle represents *Isoetes sinensis* at one specific altitude and season (WH‐low, KM‐mid, and LS‐high represent Wuhan (low altitude), Kunming (mid altitude), and Lhasa (high altitude)). Number of biased genes and other details are provided in each graph. (a–d) comparison of biased genes among three altitudes. (e–j) comparison of biased genes between summer and winter.

We first compared the *I. sinensis* that transplanted at three altitudes. In summer, 229 genes exhibited expression bias toward T subgenome, while 346 genes toward Y subgenome (Figure 2a, b). In winter, there were 598 and 623 genes, respectively, biased toward T and Y subgenome (Figure 2c, d). Whatever the season, more Y‐biased genes than T‐biased were observed, which indicated that genes from Y subgenome might play a more important role in high‐altitude adaptation. Moreover, we did a similar comparison between summer and winter. In WH‐low, 275 and 407 genes showed T‐biased and Y‐biased expression (Figure 2e, f), while the corresponding numbers were 528 and 584 in KM‐mid (Figure 2g, h), and 675 and 665 in LS‐high (Figure 2i, j), respectively. The results indicated that both T‐biased and Y‐biased genes were more shared in plateau areas (KM‐mid or LS‐high) than in low altitude area (WH‐low), which may be due to greater seasonal variations in WH‐low.

In total, 1426 and 1321 gene pairs with extreme expression bias patterns (EEBP) were detected in summer and winter, respectively (Table 3). For instance, in summer, 19 genes in WH‐low and KM‐mid showed strict bias toward the T subgenome, but no bias in LS‐high. For instance, eIF‐5a (eukaryotic translation initiation factor 5A) showed bias to the T subgenome in WH‐low and KM‐mid in winter but showed no bias in LS‐high. Another gene, Polyketide‐cyc2, showed bias toward Y subgenome in summer in WH‐low, but no bias in KM‐mid and LS‐high. Moreover, a total of 1771, 797 and 744 gene pairs with EEBP were detected in WH‐low, KM‐mid, and LS‐high, respectively (Table 4). Our results showed that *I. sinensis* transplanted in WH‐low had more gene pairs with EEBP compared with KM‐mid and LS‐high. Especially, in WH‐low, eight homoeologous gene pairs exhibited opposite bias direction, which was Y‐biased in summer but T‐biased in winter, such as Hydrolase‐4.

**TABLE 3:** Summary of homologous gene pairs with extreme expression bias patterns among three altitudes

Wuhan	Kunming	Lhasa	Counts in summer	Counts in winter
Tr > Yr	Tr > Yr	Tr > Yr	229	598
Tr > Yr	Tr > Yr	Tr < Yr	0	0
Tr > Yr	Tr > Yr	No bias	19	95
Tr > Yr	Tr < Yr	Tr > Yr	0	0
Tr > Yr	Tr < Yr	Tr < Yr	0	0
Tr > Yr	Tr < Yr	No bias	0	0
Tr > Yr	No bias	Tr > Yr	24	222
Tr > Yr	No bias	Tr < Yr	0	0
Tr > Yr	No bias	No bias	40	270
Tr < Yr	Tr > Yr	Tr > Yr	0	0
Tr < Yr	Tr > Yr	Tr < Yr	0	0
Tr < Yr	Tr > Yr	No bias	0	0
Tr < Yr	Tr < Yr	Tr > Yr	0	0
Tr < Yr	Tr < Yr	Tr < Yr	347	623
Tr < Yr	Tr < Yr	No bias	15	101
Tr < Yr	No bias	Tr > Yr	0	0
Tr < Yr	No bias	Tr < Yr	21	191
Tr < Yr	No bias	No bias	61	261
No bias	Tr > Yr	Tr > Yr	403	11
No bias	Tr > Yr	Tr < Yr	0	0
No bias	Tr > Yr	No bias	110	19
No bias	Tr < Yr	Tr > Yr	0	0
No bias	Tr < Yr	Tr < Yr	308	11
No bias	Tr < Yr	No bias	109	25
No bias	No bias	Tr > Yr	172	65
No bias	No bias	Tr < Yr	144	50
No bias	No bias	No bias	3901	3608

**TABLE 4:** Summary of homologous gene pairs with extreme expression bias patterns between seasons

Location	Summer	Winter	Counts
Wuhan	Tr > Yr	Tr > Yr	275
	Tr > Yr	Tr < Yr	0
	Tr > Yr	ns	42
	Tr < Yr	Tr < Yr	407
	Tr < Yr	Tr > Yr	8
	Tr < Yr	ns	51
	ns	Tr > Yr	885
	ns	Tr < Yr	785
	ns	ns	3693
Kunming	Tr > Yr	Tr > Yr	528
	Tr > Yr	Tr < Yr	0
	Tr > Yr	ns	244
	Tr < Yr	Tr < Yr	584
	Tr < Yr	Tr > Yr	0
	Tr < Yr	ns	207
	ns	Tr > Yr	180
	ns	Tr < Yr	166
	ns	ns	4223
Lhasa	Tr > Yr	Tr > Yr	675
	Tr > Yr	Tr < Yr	0
	Tr > Yr	ns	158
	Tr < Yr	Tr < Yr	665
	Tr < Yr	Tr > Yr	0
	Tr < Yr	ns	163
	ns	Tr > Yr	217
	ns	Tr < Yr	206
	ns	ns	3981

Furthermore, we, respectively, performed KEGG enrichment analysis of the DEGs in 12 groups which contained different seasons, altitudes, and subgenome bias, for example, Summer‐WH‐Tbias, Summer‐WH‐Ybias (see Figure S2). A total of 45 pathways were significantly enriched (*p* value ≤.05). Among all of them, 38 pathways were from Y‐biased genes, while only 7 pathways were from T‐biased genes. We further compared these pathways between different seasons and between different altitudes. Notably, for Y‐biased genes, ribosome pathway was significantly enriched in all three altitudes, phosphatidylinositol signaling system pathway was significantly enriched in KM‐mid and LS‐high (plateau areas), and photosynthesis pathway was significantly enriched in WH‐low and KM‐mid. Moreover, several unique pathways were significantly enriched in specific altitude. For example, carotenoid biosynthesis were uniquely enriched in KM‐mid. More detail information was displayed in Figure S2.

### 3.4 Uniquely expressed homoeologous genes

Uniquely expressed homoeologous genes were extracted as the crucial genes to understand the adaptation of *I. sinensis* to different altitudes. In summer, a total of 297, 137 and 49 genes were identified as unique expression in WH‐low, KM‐mid, and LS‐high, while the corresponding counts were 68, 144 and 57 in winter, respectively. The detailed information of top 5 expressed genes was shown in Table S3. The highly expressed genes were mainly associated with temperature response. For example, we detected that heat‐shock proteins (HSP20 and HSP40) had high expression level in WH‐low which were related to high temperature response. While LTP‐2, FA‐desaturase‐2 and WES‐acyltransf had high expression level in WH‐low and LS‐high, respectively, which were related to low temperature response. Moreover, we found that most of these uniquely expressed homoeologous genes, especially genes related with temperature response, showed bias toward Y subgenome (Table S3).

### 3.5 WGCNA analysis of homoeologous genes

**FIGURE 3:**
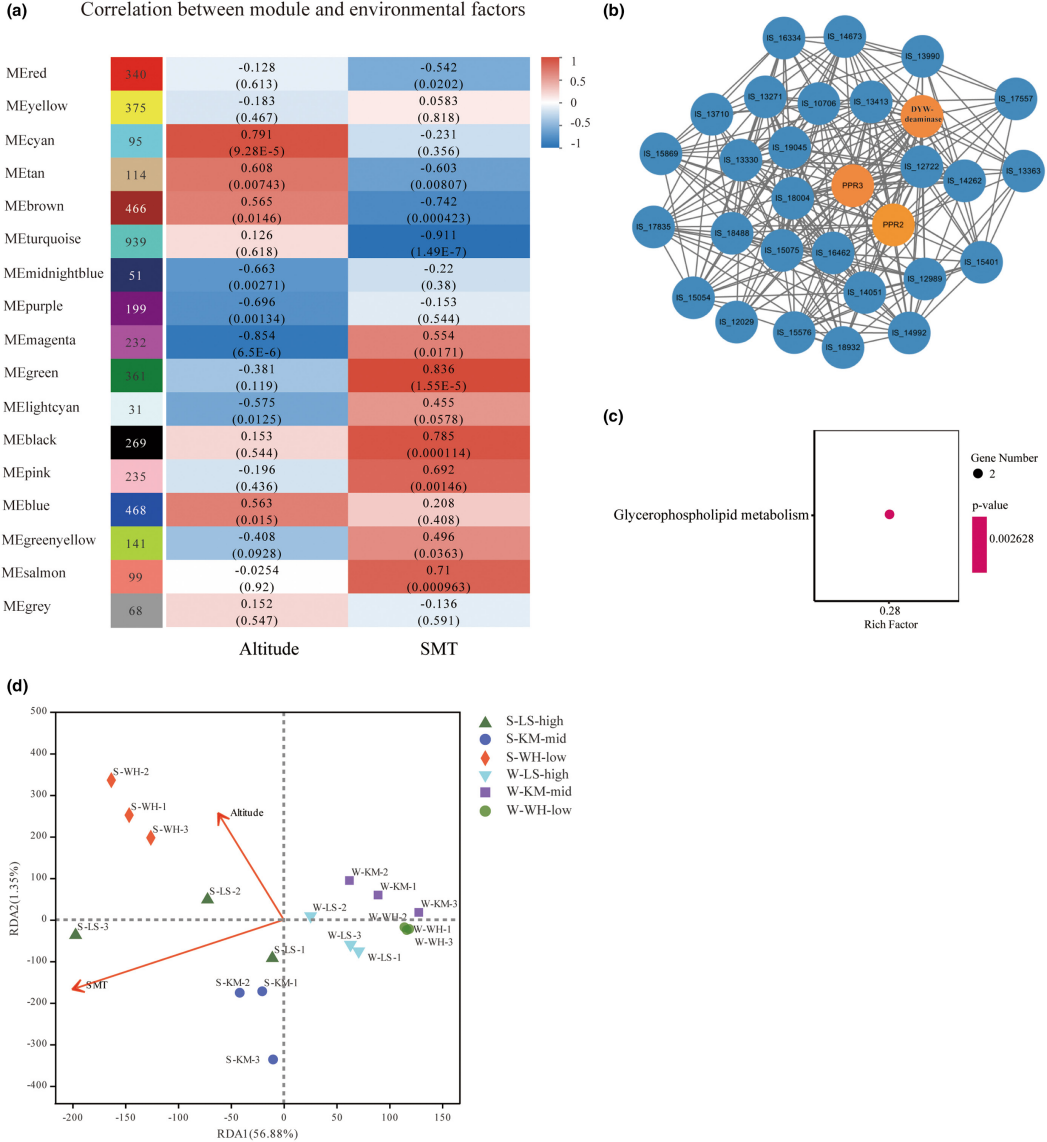
Weighted gene co‐expression network analysis and RDA analysis. (a) Module‐trait relationship of *Isoetes sinensis*. Each square is denoted the corresponding correlation coefficient and *p*‐value. SMT is the abbreviation of seasonal mean temperature. (b) KEGG enrichment analysis of genes in the cyan module. (c) Interaction network of genes in the cyan module which is the top positive module associated with altitude. Hub genes are indicated by orange circles. (d) Redundancy analysis (RDA). Symbols indicate sampling sites. Arrows indicate environmental factors.

In total, 17 modules were identified (*p* < .05) (Figure 3a). Of these, seven and 10 modules were significantly correlated with altitude and SMT, respectively. And the corresponding gene numbers were 1542 and 3196, respectively. The cyan module (95 genes) and magenta module (232 genes) were the top positive and negative modules associated with altitude. While the top positive and negative modules of SMT were the green module (361 genes) and the turquoise module (939 genes). Most of the genes in cyan module (over 76%), which were positively related with altitude, showed no bias expression.

For each top positive/negative module, three genes, which had the highest KME values, were selected as the hub genes. For altitude, the hub genes in the cyan module belonged to PPR (pentatricopeptide repeats) gene family (Figure 3b), while the two hub genes in the magenta module encoded amino acid permease family protein (AAP), glycosamine inositolphosphorylceramide transferase 1 (GINT1) and another one hub gene belonged to TIFY gene family. Meanwhile, for SMT, hub genes associated with green module encoded fasciclin‐like arabinogalactan proteins (FLAs), eucine‐rich repeat receptor‐like protein kinase (LRR‐RLKs) and pectin lyase‐like superfamily protein (PPME), while the hub genes in turquoise module encoded RNA helicase family protein, protein translocase subunit SECA2 and coiled coil protein. The KEGG enrichment analysis of these genes showed that the glycerophospholipid metabolism pathway was significantly enriched based on the genes in the cyan module (Figure 3c). No significant KEGG pathway was identified in the magenta module.

From a quantitative point of view, gene expression may be more affected by seasonal mean temperature. The result of redundancy analysis (RDA) indicated that both altitude and SMT contributed variously to gene expression (Figure 3d). As expected, SMT had more contribution, indicating that it played a more important role in the spatiotemporal variations of transcriptome.

## 4 DISSCUSSION

Polyploids are characterized by increased genomic content and genome size, which potentially provide strong tolerance to environmental stresses and increase the possibility to colonize new habitat. Previous studies of plant polyploidization support the potential for increased adaptability in polyploid lineages (Brochmann et al., 2004; Levin, 1983; Otto & Whitton, 2000; Van De Peer et al., 2021). We assumed that allopolyploid *I. sinensis* inherited the potential of adapting to plateau from its high altitude adapted parents *I. yunguiensis*. To test the hypothesis, we design the transplanting experiment. One year after transplanting, it still lived well, even in the extreme environment of the Qinghai‐Tibet Plateau. Through comparative transcriptome analysis, we measured the contribution of T and Y subgenomes to environmental adaptation. In conclusion, the allopolyploid *I. sinensis* is adaptive to plateau and the potential of adapting to plateau may inherit from its high altitude adapted progenitor.

### 4.1 Homoeologous gene expression pattern in response to altitude and season

Until now, almost no study has been reported about the homoeologous gene expression bias of allopolyploid in genus *Isoetes*. For the published homoeologous gene expression studies, most of them focus on some cash‐crop, such as *Coffea* (Combes et al., 2013), *Brassic* (Li et al., 2020), and cotton species (Mei et al., 2021). A few researches have been addressed on the impact of temperature on subgenome expression bias (Combes et al., 2012), but almost all of them were control experiments that cannot represent the actual condition in natural environments. To explore the relationship between homoeologous gene expression bias and altitude/season, we transplanted *I. sinensis* to gradient altitudes and performed transcriptome sequencing. The statistics tables of homoeologous gene expression bias were provided in Tables 1 and 2. We further analyzed the influence of altitude and season on the expression bias of homoeologous gene. This is an exploratory and original study that can enrich our understanding of the influence of environmental factors on the expression pattern of homologous genes.

Both summer and winter are the two most extreme seasons of 1 year. However, no change in subgenome bias direction was observed in the comparison of the two seasons (Table 2). While this change occurred between different altitudes, with bias to Y, Y and T subgenome in WH‐low, KM‐mid, and LS‐high, respectively. Therefore, we speculate that the seasonal variations cannot change the subgenome bias trend of homoeologous gene pairs in *I. sinensis*, but the altitude can. This may be because the altitude is a more comprehensive and complex factor that has diverse effects on plants, such as plant trait variation (Cui et al., 2018; Giupponi et al., 2020; Tsegay et al., 2020) and plant genetic variation (Byars et al., 2009; Hahn et al., 2012).

### 4.2 Uniquely expressed genes are mainly related to temperature response

Changes in environmental conditions, such as light environment, temperature, or water status, may lead to altered gene expression in plants. In short‐term adaptation, organisms improve their adaptive ability mainly by generating suitable chemical compounds to adapt to rapidly changing environments (Ho & Zhang, 2018). Heat stress is one of the most important environmental stress (Wahid et al., 2007). High temperature may cause adverse effects on photosynthesis, respiration, membrane stability, and water relations, and enhance the expression of a series of heat shock proteins and promote the production of reactive oxygen species (ROS) (Wahid et al., 2007). In this study, we identified several uniquely expressed genes which were associated with high‐temperature response. In summer, 1‐cysPrx‐C, HSP20, and DnaJ were identified as highly expressed in WH‐low (SMT of summer: 27.99°C). 1‐cysPrx‐C is a thiol‐dependent antioxidant enzyme. 1‐cysPrx‐C is concentrated in the nucleus and involved in protecting nucleic acids from ROS (Mowla et al., 2002). HSP20 is the most abundant class among the HSP group (Heckathorn et al., 1999). HSP20 can bind to the newly synthesized and denatured proteins to preventthe formation of irreversible aggregations (Zhang et al., 2015). While, DnaJ is another class of HSP, known as heat‐shock protein 40 (HSP40). It performs the function of molecular chaperones independently or as the co‐chaperone of HSP70 (Craig et al., 2006; Hennessy et al., 2005). Previous study of *Solanum lycopersicum* had demonstrated that DnaJ protein could enhance thermotolerance by maintaining Rubisco activity (Wang et al., 2015). HSPs play a role in stabilizing cell membranes and proteins, and help proteins refold under stressful conditions (Wang et al., 2004). The production of HSPs is an essential process in response to high temperature (Vierling., 1991). SLAC1 (slow anion channel‐associated 1), which can induce stomatal closure, was highly expressed in KM‐mid (SMT of summer: 20.18°C). Closing stomata is a common response of plants to abiotic stress (Ainsworth, 2017). Plants can reduce water loss through stomatal closure to improve heat tolerance and drought resistance. SLAC1 preferentially expresses in guard cells and is essential for stomatal closure in response to multiple environmental stresses (Vahisalu et al., 2008). The temperature of LS‐high in summer is relatively low with a SMT of 14.33°C. Thus, *I. sinensis* may not be subjected to heat stress so that none of these 57 uniquely expressed genes involved the response to heat stress, but some other regulations.

Cold stress is another important stress that can prevent the expression of genetic potential. Cold stress can directly inhibit the metabolic reactions (Chinnusamy et al., 2007). Cold stress related genes were highly expressed in WH‐low and LS‐high (SMT of winter: 5.69°C and −1.84°C; extremely low temperatures: −12.8°C and −23.1°C, respectively), but almost none in KM‐mid (SMT of winter: 9.28°C; extremely low temperatures: −3°C), which might be due to the cold winters of WH‐low and LS‐high. The mobility and stability of cellular membrane is an important factor for plant cells (Li et al., 2018). However, cold temperatures can reduce the fluidity of cellular membranes and affect their basic functions (Chinnusamy et al., 2007). We detected that LTP‐2 (lipid transfer protein 2) exhibited high expression in WH‐low, which helps stabilize membranes during freezing (Hincha, 2002). Other highly expressed genes in WH‐low are mainly related to plant defense and low‐temperature response. While, in KM‐mid, genes related to modulates photomorphogenesis and photoperiod response exhibited high expression. LS‐high locates on the Qinghai‐Tibetan Plateau, which is characterized by strong solar radiation and extreme low temperature (Mao et al., 2021). More highly expressed genes were related to chilling tolerance, such as FA‐desaturase‐2 and WES‐acyltransf. Morever, most of these uniquely expressed genes showed bias toward Y subgenome, indicating that Y subgenome provided more contributions to high altitude adaptation, especially for temperature response. The *I. yunguiensis*, one of the progenitors of *I. sinensis*, are distributed in high altitude area (YGP). We thus speculate that *I. sinensis* retains the characteristics inherited from its parent *I. yunguiensis* during its long evolutionary history, and these potential characteristics manifested in the process of retransplantation to high altitude areas.

### 4.3 More KEGG pathways are significantly enriched in Y‐biased genes

From the KEGG enrichment analysis results of DEGs, Y subgenome played a more important role in adaptation to different altitudes. Our study identified several important pathways for altitude adaptation, which were enriched in Y‐biased homoeologous genes. Ribosomes are vital organelles for protein synthesis (Wilson & Doudna Cate, 2012). The important role of ribosomal proteins in response to various abiotic stressors has previously been demonstrated (Kosová et al., 2018; Salih et al., 2020). In this study, ribosome pathway was significantly enriched in all six groups. The expression of abundant ribosome‐related genes enabled *I. sinensis* to make effective adjustments in metabolism and development (Wang et al., 2013). Photosynthesis is a fundamental and essential biological process of plants (Wu et al., 2008). The homoeologous genes related to photosynthesis exhibited significant differential expression in WH‐low and KM‐mid, but not in LS‐high. This is consistent with previous findings from our laboratory that UV radiation is the major stressor at high altitude (QTP) and can cause photosynthesis suppression (Dai et al., 2021). Besides, we found that phosphatidylinositol (PI) signaling system pathway was only significantly enriched in plateau areas (KM‐mid and LS‐high) in both summer and winter. PI signaling system is widely involved in the response of organisms to environmental factors (Carafoli, 2002), and involved in multiple developmental and physiological pathways (Dove et al., 1997; Mueller‐Roeber & Pical, 2002; Perera et al., 1999). Our result indicated that PI signaling system might play an important role in high altitude adaptation of *I. sinensis*.

### 4.4 Hub genes and key pathway in altitude adaptation‐related module

WGCNA analysis detected 1542 and 3196 genes that were significantly correlated with altitude and SMT, respectively. From which, a total of 12 genes were identified as hub genes. It is worth noting that the top 3 genes of cyan module (sorted by KME) were all belonging to PPR gene family. PPR proteins are encoded by nuclear genome, but they are mostly targeted to plastids and mitochondria (Colcombet et al., 2013). PPR protein binds to organellar transcripts, and affects their expression by altering RNA sequence, processing, turnover, or translation (Barkan & Small, 2014). Previous research indicated that PPRs are involved in a wide range of events, including physiological and developmental processes and response to various biotic and abiotic stresses (Xing et al., 2018). There was also study suggested that PPR proteins govern a series of functions in organelles genomes, including the stabilization of organelles transcripts, RNA editing and the fertility restoration of CMS lines (Kaur & Verma, 2016). The high UV‐B radiation at high altitudes can cause damage to plants, especially in QTP. The RNA editing of PPRs can mitigate DNA damage from increased UV exposure (Yan et al., 2018). The expression of PPR genes showed a positive correlation with altitude, indicating that PPR genes may have an important role in adaptation to high‐altitude UV radiation.

Temperature shows some dependency on altitude. Generally, higher altitude regions are colder than low altitude regions. That is, high‐altitude plants are more cold‐stressed than low‐altitude plants. In addition to cold stress, plants will confront a variety of problems, such as low oxygen and high UV radiation (Kofidis et al., 2003). In the long‐term evolution process, plants have developed complex signaling mechanisms to deal with environmental stress (Ning et al., 2010). A lot of signal molecules have been well studied. In our study, Glycerophospholipid (GPL) metabolism pathway was significantly enriched in the cyan module, which has a strong positive relationship with altitude. GPLs are from the cell membranes and have diverse functions (Sosa Alderete et al., 2020). GPLs provide energy for cellular metabolism, and are a rich source for signal molecules synthesis that involves a wide variety of cellular processes such as development, and response to adverse environmental conditions. For example, cold and frost induce the formation of phosphatidic acid (PA) via both phospholipase D (PLD) and diacylglycerol kinase (DGK) pathways (Ruelland et al., 2002). And the previous study of *Arabidopsis thaliana* indicated that PA formed via AtPLDdelta action helped the plant to acclimate to cold stress (Katagiri et al., 2001), suggesting that GPL may play an important role in adaptation to high‐altitude cold environments.

## AUTHOR CONTRIBUTIONS

**Pei Wei:** Data curation (lead); formal analysis (lead); methodology (lead); writing – original draft (lead). **Xiao‐lei Yu:** Writing – review and editing (lead). **Yu‐jiao Yang:** Methodology (supporting). **Zhu‐yifu Chen:** Formal analysis (supporting). **Shu‐qi Zhao:** Data curation (supporting). **Xin‐zhong Li:** Investigation (supporting). **Wen‐cai Zhang:** Investigation (supporting). **Chen‐lai Liu:** Formal analysis (supporting). **Xiao‐yan Li:** Supervision (supporting). **Xing Liu:** Writing – review and editing (lead).

### OPEN RESEARCH BADGES

This article has earned an Open Data badge for making publicly available the digitally‐shareable data necessary to reproduce the reported results. The data is available at https://www.ncbi.nlm.nih.gov/bioproject/PRJNA857183.

## Supporting information


Table S1

Table S2

Table S3

Figure S1

Figure S2

## Data Availability

The raw sequencing data has been deposited in the National Centre for Biotechnology Information (NCBI) under accession number SAMN29620894–SAMN29620902.
