# Supplementary material for: Biased gene expression reveals the contribution of subgenome to altitude adaptation in allopolyploid Isoetes sinensis
Source: Ecol Evol. 2022 Dec 28;12(12):e9677. doi: 10.1002/ece3.9677 (PMC9797765; doi:10.1002/ece3.9677)
Supplement: Supplementary file 1 — Table S1 [file ECE3-12-e9677-s005.docx]

Table S1 Seasonal mean temperature

| **Season** | **Site** | **Seasonal mean temperature(℃)** |
| --- | --- | --- |
| **Summer** | Wuhan | 27.99 |
|  | Kunming | 20.18 |
|  | Lhasa | 14.33 |
| **Winter** | Wuhan | 5.69 |
|  | Kunming | 9.28 |
|  | Lhasa | -1.84 |
