# Supplementary material for: Biased gene expression reveals the contribution of subgenome to altitude adaptation in allopolyploid Isoetes sinensis
Source: Ecol Evol. 2022 Dec 28;12(12):e9677. doi: 10.1002/ece3.9677 (PMC9797765; doi:10.1002/ece3.9677)
Supplement: Supplementary file 2 — Table S2 [file ECE3-12-e9677-s002.docx]

Table S2 Statistics of RNA-seq data

| **Season** | **Samples** | **Raw reads (Million)** | **Clean reads (Million)** | **Q20 (%)** | **Q30 (%)** | **Uniquely mapping to genome ratio (%)** | **GC content (%)** |
| --- | --- | --- | --- | --- | --- | --- | --- |
| **Summer** | Wuhan1 | 51.74 | 50.77 | 97.28 | 92.91 | 65.24 | 46.33 |
|  | Wuhan2 | 48.66 | 47.74 | 97.26 | 92.89 | 65.92 | 46.08 |
|  | Wuhan3 | 54.81 | 53.51 | 96.99 | 92.29 | 68.46 | 45.53 |
|  | Kunming1 | 43.66 | 43.04 | 96.91 | 91.88 | 63.33 | 45.38 |
|  | Kunming2 | 43.45 | 42.48 | 96.79 | 91.80 | 61.27 | 46.65 |
|  | Kunming3 | 50.94 | 50.39 | 98.61 | 96.14 | 62.08 | 46.00 |
|  | Lhasa1 | 51.76 | 50.61 | 96.80 | 91.83 | 62.79 | 45.85 |
|  | Lhasa2 | 42.79 | 41.99 | 97.00 | 92.27 | 64.56 | 45.84 |
|  | Lhasa3 | 47.09 | 46.18 | 96.97 | 92.20 | 64.57 | 46.85 |
| **Winter** | Wuhan1 | 50.87 | 50.66 | 97.96 | 93.85 | 61.85 | 44.29 |
|  | Wuhan2 | 43.88 | 43.67 | 97.90 | 93.71 | 62.67 | 44.38 |
|  | Wuhan3 | 40.09 | 39.92 | 97.87 | 93.62 | 60.73 | 44.24 |
|  | Kunming1 | 41.27 | 41.04 | 97.71 | 93.25 | 63.63 | 44.22 |
|  | Kunming2 | 40.88 | 40.69 | 97.92 | 93.74 | 63.02 | 44.22 |
|  | Kunming3 | 41.26 | 41.05 | 97.70 | 93.23 | 59.21 | 44.60 |
|  | Lhasa1 | 50.59 | 50.32 | 98.01 | 94.17 | 64.99 | 44.22 |
|  | Lhasa2 | 45.01 | 44.81 | 97.96 | 94.07 | 64.40 | 45.09 |
|  | Lhasa3 | 46.11 | 45.83 | 97.95 | 94.04 | 63.60 | 44.09 |
