# Supplementary material for: Biased gene expression reveals the contribution of subgenome to altitude adaptation in allopolyploid Isoetes sinensis
Source: Ecol Evol. 2022 Dec 28;12(12):e9677. doi: 10.1002/ece3.9677 (PMC9797765; doi:10.1002/ece3.9677)
Supplement: Supplementary file 3 — Table S3 [file ECE3-12-e9677-s004.docx]

Table S3 Top 5 uniquely expressed homologous genes

| **Season** | **Location** | **Gene or product name** | **Bias toward** |
| --- | --- | --- | --- |
| **Summer** | Wuhan | HSP20, 1-cysPrx-C, DnaJ, Rad21, Oleosin | Y, Y, Y, Y, N |
|  | Kunming | Rad51, SLAC1, GASA, Polyketide-cyc2, EF-hand 5 | T, N, N, T, T |
|  | Lhasa | Glyco-hydro-28, FMN-dh, NifU, ABC-tran, DNA methylase | Y, N, Y, Y, Y |
| **Winter** | Wuhan | PAR1, LTP-2, PAP2-C, Frataxin CyaY, DPY-30 | T, Y, N, T, T |
|  | Kunming | BSP, S6PP, JAB, Dirigent, Mlo | Y, Y, Y, Y, N |
|  | Lhasa | FA-desaturase-2, WES-acyltransf, AP2, DUF1929, RRM-1 | Y, Y, N, T, Y |
