# Supplementary figures and images for: Biased gene expression reveals the contribution of subgenome to altitude adaptation in allopolyploid Isoetes sinensis
Source: Ecol Evol. 2022 Dec 28;12(12):e9677. doi: 10.1002/ece3.9677 (PMC9797765; doi:10.1002/ece3.9677)

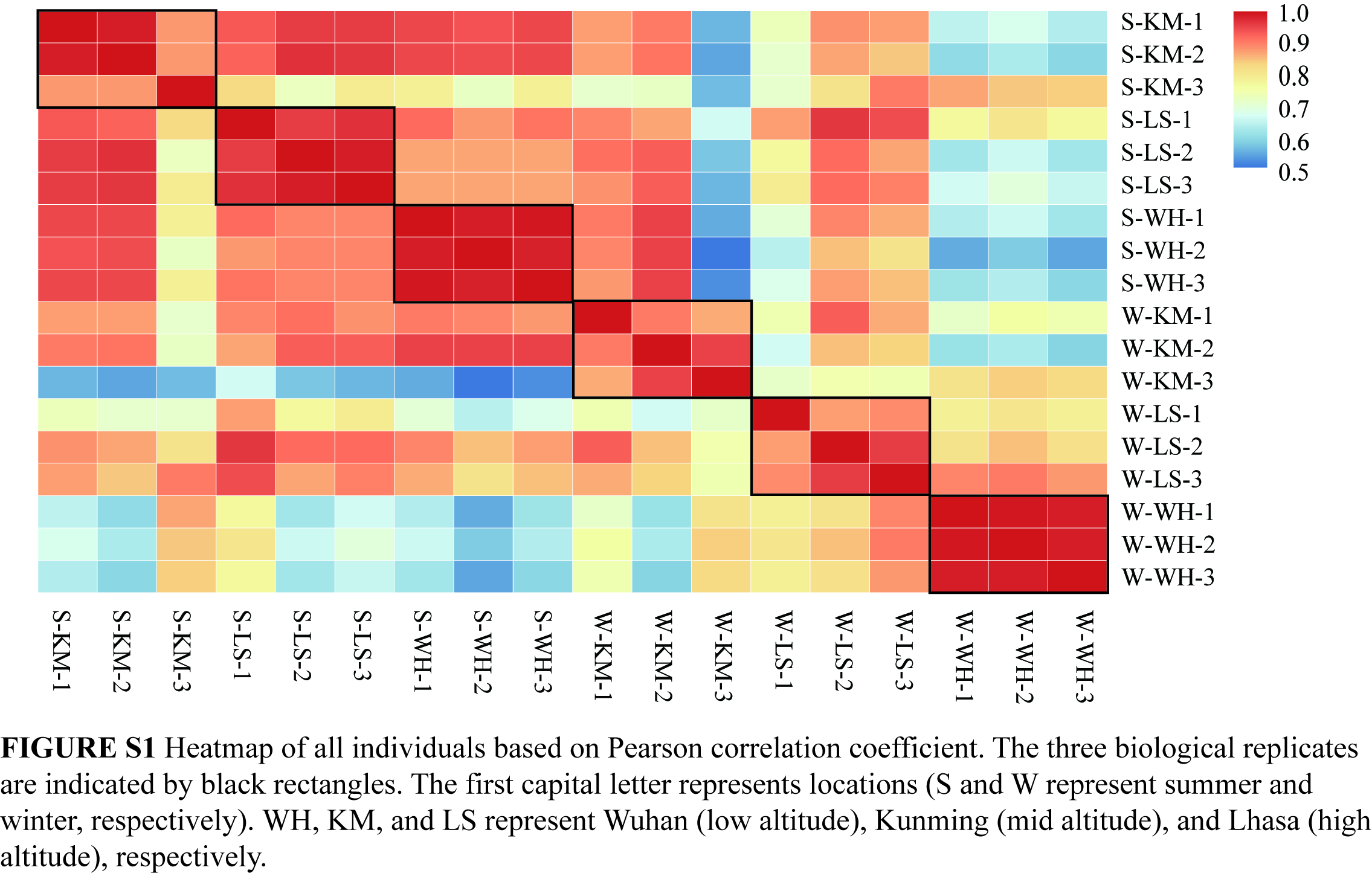

Supplement: Supplementary file 4 — Figure S1 [file ECE3-12-e9677-s001.tif]

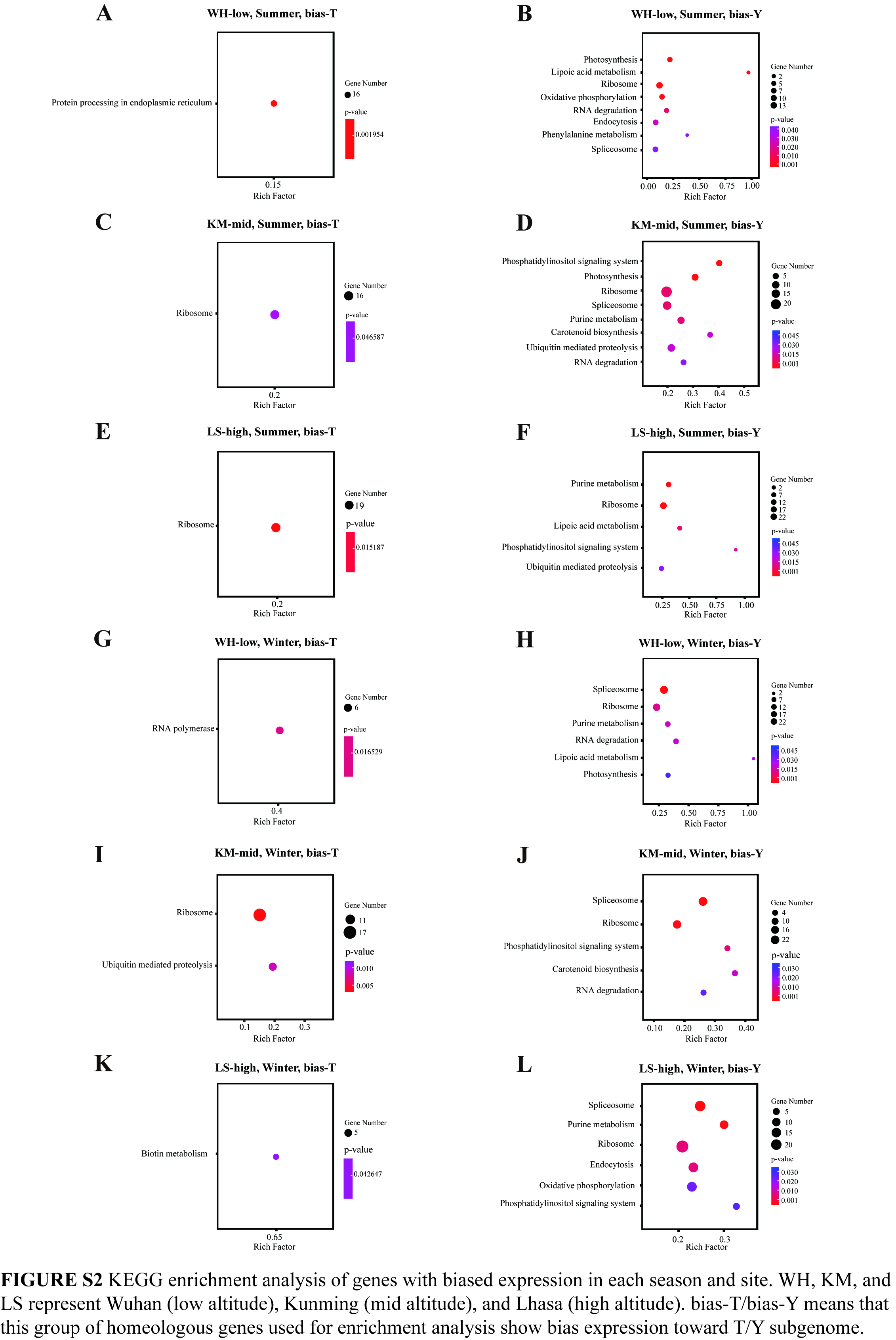

Supplement: Supplementary file 5 — Figure S2 [file ECE3-12-e9677-s003.tif]
